# Assessing organophosphate insecticide retention in muscle tissues of juvenile common carp fish under acute toxicity tests

**DOI:** 10.1016/j.toxrep.2024.02.002

**Published:** 2024-02-10

**Authors:** Imtiyaz Qayoom, Masood Balkhi, Malik Mukhtar, Adnan Abubakr, Uzma Siddiqui, Sameena Khan, Asma Sherwani, Ishrat Jan, Riyazali Sayyed, Andrea Mastinu

**Affiliations:** a Division of AEM, Faculty of Fisheries, Sher-e-Kashmir University of Agricultural Sciences and Technology of Kashmir (SKUAST-K), Rangil Ganderbal, Jammu and Kashmir 191201, India; b Division of Entomology, Faculty of Horticulture, Sher-e-Kashmir University of Agricultural Sciences and Technology, Shalimar, Srinagar, Jammu and Kashmir 190025, India; c Department Zoology, DSB-Campus Kumaun University, Nainital 263001, India; d Research Centre for Residue and Quality Analysis, Sher-e-Kashmir University of Agricultural Sciences and Technology (SKUAST-K), Shalimar Campus, Srinagar 190025, India; e Department of Microbiology, PSGVP Mandal’s S I Arts, G B Patel Science and STKV Sangh Commerce College, Shahada 425409, India; f Faculty of Health and Life Sciences, INTI International University, 71800 Nilai, Negeri Sembilan, Malaysia; g Department of Molecular and Translational Medicine, Division of Pharmacology, University of Brescia, 25123 Brescia, Italy

**Keywords:** Bioaccumulation, Chlorpyrifos, Dimethoate, *Cyprinus carpio*, Toxicity, Bio-concentration factor

## Abstract

Organophosphate insecticide spray poses potential threat of contamination of environmental components their accumulation in aquatic organisms. Although various physiological deficits associated with their exposure in fishes are documented, yet their retention in their edible muscle tissues has been poorly studied. In this context, the study was undertaken to ascertain the bioaccumulation of two organophosphate insecticide compounds (dimethoate and chlorpyrifos) in the muscles of juvenile *Cyprinus carpio*. The study could provide insight into the risks to human health associated with consuming contaminated fish flesh. The fishes exposed to various concentrations of dimethoate and chlorpyrifos in-vivo for 96 to ascertain the uptake and retention of these insecticides in the muscle. Results indicated that fish muscles accumulated the residues at all the concentrations with the recovery of 2.99% (0.032 ppm) of dimethoate exposed to LC_50_ concentrations. In contrast, the chlorpyrifos residues were found Below the Detection Level (BDL) in the fishes exposed to LC_50_ concentrations. The percentage bioaccumulation of dimethoate in fish muscle was 88.10%, and that of chlorpyrifos was BDL. The bio-concentration factor was dose-dependent and increased with increasing doses of both insecticides. The study invites attention to human health risk assessment in the regions where contaminated fish are consumed without scientific supervision.

## Highlights

• Organophosphate insecticides accumulate in muscle and other tissues of *Cyprinus carpio*.
• It exerts fatal effects on *Cyprinus carpio,* aquatic ecosystem and food safety.
• Insecticide retention analysis reveals the hazards of pesticide use in aquatic ecosystems.
• The data entails the need for pesticide management in aquatic ecosystems.
• Since this fish is consumed globally, insecticide residues in fish reflects food safety concerns.

## Graphical Abstract

**Figure:**
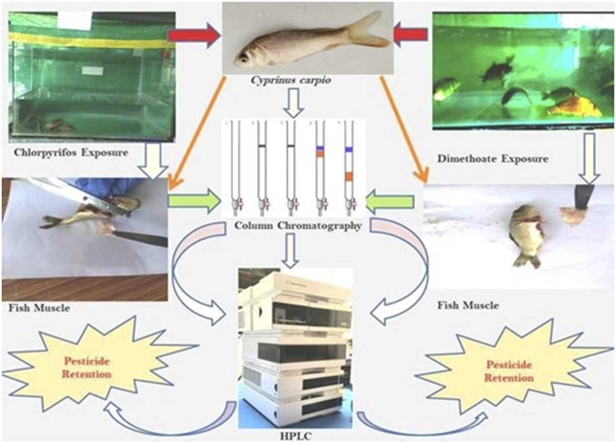

## 1 Introduction

Environmental pollution by insecticides has become one of the most significant issues worldwide [7]. Chemical products discharged into the environment reach aquatic systems, contaminating and affecting the marine biota, including fishes, through direct contact of the body surface and the gills of these animals with contaminated water or else through their food. Upon entering the fish body, insecticides result in several clinical changes in their behaviour, physiology, or genetic makeup within the short exposure period. In addition to the other physiological dysfunctions, fish can bio-accumulate insecticide residues manyfold than the surrounding water [35], which, if subsequently consumed by human beings, could prove hazardous to them [28]. Therefore, it becomes imperative to ascertain the levels of insecticide contamination in natural water bodies and commercially important plants and animals inhabiting them.

Identification and quantification of insecticide residues from fish muscles form the primary tool of toxicological risk assessment, which proves helpful in determining the level of penetration of the insecticides into the muscle tissues and the quality of fish for human consumption. The residue analysis of insecticides can be carried out in fish samples collected either from the natural waterbodies or from the experimental animals subjected to in-vitro insecticide exposure. The former method can be implied as part of the environmental monitoring assessment of aquatic ecosystems for the hazard analysis and calculation of different insecticides' Maximum Residue Level (MRL) values. At the same time, the latter can be used mainly for monitoring other physiological indices and the bio-concentration factor of chemicals in aquatic organisms.

Dimethoate [O,O-dimethyl S-methyl-carbamoyl-methylphosphorodithioate] and chlorpyrifos [O,O-Diethyl-O-(3,5,6-trichloro-2- pyridinyl) phosphonothioate](Scheme 1) are the broad spectrum cholinergic organophosphates known as acetylcholinesterase (AChE) inhibitors [1], [3]. Despite being characterized as an organophosphate compound, chlorpyrifos is typically known to have lipophilic properties due to three chlorine atoms in the parent benzene ring. For this reason, it depicts the lipophilic properties more or less like chlorinated hydrocarbons. The persistence of chlorpyrifos (36.9 days) is similar to that of endosulfan (alpha endosulfan = 20.4 days; beta endosulfan = 63.6 days) in a water/sediment microcosm study under tropical conditions [21] and therefore both are regarded as moderately persistent compounds. Dimethoate, on the other hand, although highly water soluble [12], is highly toxic to fishes and other aquatic invertebrates [1].

With this background, the current study aimed to evaluate the retention of chlorpyrifos and dimethoate residues in the muscles of *Cyprinus carpio* var. *communis* juveniles after they were exposed to short-term exposure for 96 h. The study will help correlate the levels of insecticides in natural waters and their bioaccumulation in fishes and help determine whether they are suitable for human consumption. Common carps have worldwide consumer preference and is consumed by large group of people. Moreover, they are emerging as conducive experimental animals due their hardy nature and ability to acclimate laboratory conditions.

## 2 Material and methods

### 2.1 Study area, procurement of samples, and acclimatization

Juvenile common carps, *Cyprinus carpio* var. *communis* (10 ± 2 g) were taken as test organisms and procured from National Fish seed Farm, Manasbal, District Ganderbal J&K India. Fishes and the aquaria (60 ×30×40 cm) keeping them were both disinfected by giving dip bath in 0.05% KMnO_4_ solution. During the acclimation period, fishes were fed with artificial diet for two weeks that was shipped from the Manasbal National Carp Farm and given @ 3% body weight once in the evening. The leftover debris (feed or faeces) was thoroughly siphoned, and the water in the tank was replaced every morning. Before the start of the experiments, feeding was stopped before 24 h and range finding tests were carried out followed by the definitive tests for the determination of median lethal concentrations.

### 2.2 Chemicals and reagents

In the present investigation, dimethoate and chlorpyrifos were used with the purity of 90% and 95.0%, respectively, procured from Hyderabad Chemicals Private Ltd. Stock solution of the insecticides were prepared in Methanol and subsequent dilutions in de-ionized water. The dimethoate and chlorpyrifos reference standard (purity 99.5% and 99.0%, respectively) was purchased from Dr. Ehrenstorfer (Augsburg), Germany. Analysis of the technical grade insecticides revealed only compounds of interest, and none of the co-eluting compounds were detected at the same retention time as the compounds being analysed. Potassium permanganate (KMnO_4_)_,_ sodium sulphate (Na_2_SO_4_) anhydrous, florisil was purchased from Sigma-Aldrich, USA. Acetonitrile [high-performance liquid chromatography (HPLC) grade], petroleum ether, and hexane were purchased from Merck, Darmstadt, Germany. The sorbents were activated in a muffle furnace at a temperature of 200 °C for 2 h before use.

### 2.3 Standard solution of insecticides

**Fig. 1:**
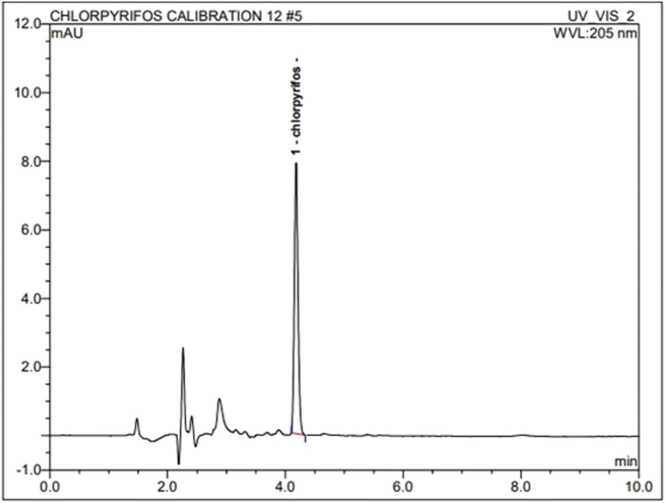
Standard of Chlorpyrifos.

**Fig. 2:**
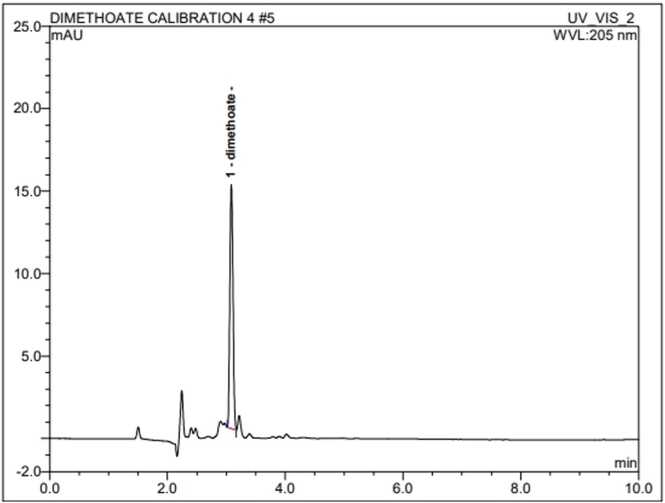
Standard of Dimethoate.

**Fig. 3:**
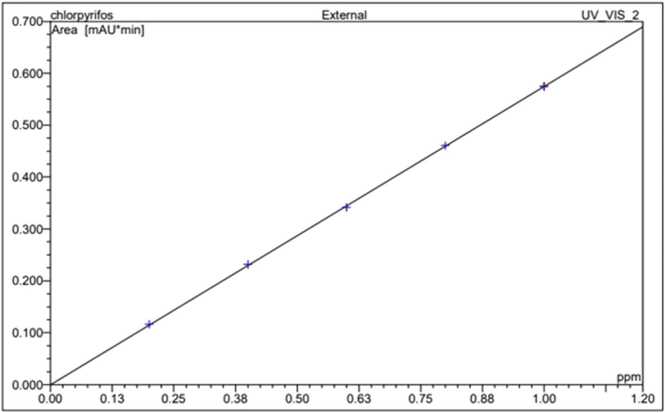
Calibration curve of Chlorpyrifos.

**Fig. 4:**
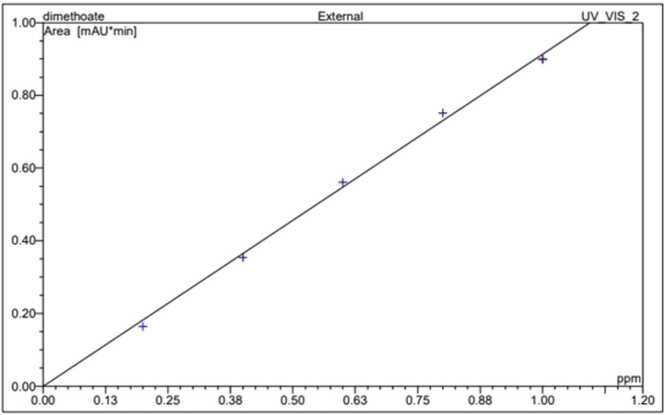
Calibration curve of Dimethoate.

Chlorpyrifos and dimethoate stock solution (1 mg ml^−1^) were prepared in HPLC-grade acetonitrile (Fig. 1, Fig. 2). The calibration curve of chlorpyrifos and dimethoate was constructed from various concentrations (0.1, 0.3, 0.5, 0.7, and 1.0 μg ml^−1^) prepared from stock solution by serial dilution with acetonitrile (Fig. 3, Fig. 4). All standard solutions were stored at 4 °C till use.

### 2.4 Toxicity tests

Toxicity tests were carried out in the Division of Aquatic Environmental Management, Faculty of Fisheries, Rangil, SKUAST-Kashmir, as per the method described by [32]) and OECD [26]. The median lethal concentrations of chlorpyrifos and dimethoate technical were chosen based on literature [29], [30], which was done on the species of the exact weight groups (8 ± 10 gm). After exposure to insecticides for 96 h, their penetration and retention in the edible muscle tissues were studied.

Double distilled water was used as the test medium in aquaria. A glass rod was then used to stir the water after the toxicant had been added to ensure that it was evenly and quickly distributed throughout the aquarium. Ten fish were put in each aquarium to ensure their freedom of movement and prevent any stress brought on by overcrowding or suffocation, and the maximum loading of the test organism was 1 gl^−1^. To ensure that the dissolved oxygen concentration at 26 °C did not exceed 6 mg L^−1^ before the experiment, the dilution water was forcefully aerated with a glass rod.

Dimethoate concentrations of 0.5, 1.0, 2.0, 4.0, and 8.0 gmL^−1^ were chosen for the definitive trial. Concentrations were chosen from the range finding tests and mean lethal concentrations were calculated from these concentrations. Likely for chlorpyrifos, decisive tests were conducted at concentrations of 4.0, 8.0, 16.0, 32.0, and 64.0 ngmL^−1^, and the mean lethal concentration was calculated using mortality estimations.

### 2.5 Insecticide residue analysis from edible muscles of fishes

In the pesticide Residue Analysis Laboratory of the Research Centre for Residues and Quality Analysis, SKUAST-Kashmir, Shalimar, pesticide residue analysis was completed. For residue analysis, fish exposed to various pesticide doses in a bioassay were utilised [33]. Only the fish's edible parts (muscle tissues), which were transported to the lab in plastic bags, were employed for the investigation. 50 g of the edible portion of the fish was taken as a laboratory sample, and the whole sample was chopped and blended in a blender (Robot Coupe Blixer 6 v.v) and homogenized in a silent crusher homogenizer (Heidolph Germany). Only 20 g of the homogenised sample from the thoroughly ground muscle section was used as a test sample in a motor. The homogenised sample was then mixed with 40 g of anhydrous sodium sulphate that had been soaked with petroleum ether for 20 min .100 ml of petroleum ether was combined with the samples and extracted and then extracted again for two minutes. The samples were then centrifuged on Q-sep 3000 (Restek, USA) centrifuge (4000 rpm) for 4 min for phase separation. The extract was decanted to a 250 ml volume flask through the 20 g anhydrous sodium sulphate bed. Insecticides adhered to the supernatant after leaving the muscle tissue and were entirely separated during centrifugation. To confirm that all pesticides had been completely removed from the muscle, the samples were once more combined with 100 ml of petroleum ether before the supernatant was collected.

### 2.6 De-fattening of fish sample extraction

A 500 ml separating funnel was used to transfer the sample extract from the flask to the funnel by mixing with 100 ml of 1:1 (v/v) n-hexane/acetonitrile solutions. Phase separation of the organic solvents was carried before letting it to stand for 20 min. Fat containing hexane solvent was discarded together with the acetonitrile fraction that contained pesticide residues. Using 25 ml of n-hexane, the resulting acetonitrile solvent extract was further cleaned up. In a rotating vacuum evaporator set at 40 °C, the acetonitrile extract was concentrated to a volume of 5 ml.

### 2.7 Clean up in Florisil Column Chromatography

A glass column with a 10 mm ID was filled with activated florisil (4 g) and around 2 cm of anhydrous sodium sulphate. To settle the adsorbent, the stop cock was opened and the column was tapped.25 cc of petroleum ether was added after the column was designated at 1 cm above the sodium sulphate layer. As soon as the solvent level reached the target, a receiving flask was positioned beneath the column. Using a micropipette, the sample extract was delivered to the column. 3 cc of petroleum ether was used to clean the sample extract flask. The solvent level could not go below the threshold, and this was carefully monitored. Oil ether (5 ml) was used to elute the column. The extract was concentrated near dryness in a rotary evaporator, reconstituted in 1 ml acetonitrile, and transferred to HPLC autosampler vials for analysis. Before HPLC analysis, all samples were filtered through a Polytetrafluoroethylene (PTFE) syringe filter (0.22 µm, Whatman’s UK).

### 2.8 HPLC Parameters

The residues of dimethoate and chlorpyrifos were quantified using High-Performance Liquid Chromatography (HPLC) coupled with a photo-diode array (PDA) detector. The HPLC was made by Dionex and has the following components: column compartment TCC-3000 SD, photo-diode array detector DAD-3000, autosampler ACC- 3000 T, and quaternary pumps LPG-3400 SD. The C18 column (Phenomenexkinetex 250 4.6 mm, 5 m, 100 A) was used for the separation. Acetonitrile and water (9:1 v/v) were used as the mobile phase, with UV detection taking place at 205 nm and a flow rate of 1 ml min^−1^. Column compartment temperature was kept at 25 °C. The total run time was 10 min. Under these operating conditions, dimethoate and chlorpyrifos retention times were 3.05 and 4.23 min, respectively. Data analysis was performed by chromeleon software 6.80 version.

The following formula quantified the quantification of insecticides recovered from the muscles of the fish:

$\mathrm{Insecticide}(\mathrm{ppm})\frac{\mathrm{Standard injected}(\mathrm{ng})}{\mathrm{Standar area}}x\frac{\mathrm{Sample area}}{\mathrm{Sample injected}(\mathrm{µl})}x\frac{\mathrm{Sample volume}}{\mathrm{Sample weight}(g)}$

Bio-concentration factor (BCF) was calculated by the following formula [15].

$\mathrm{BCF}(\mathrm{L per}\mathrm{kg})\frac{\mathrm{The concentration of the substance in fish}(\mathrm{mg per kg})}{\mathrm{The concentration of the substance in water}(\mathrm{mg per L})}$

## 3 Experimental findings

### 3.1 Insecticide fortification and recovery

The limit of instrument detection (LOD) was 0.01-μg g^−1^and limit of quantification (LOQ) for dimethoate and chlorpyrifos in fish muscles was found to be 0.03-μgg^−1^. Recovery experiments were carried out to check the in-house validity of the method in fish muscle by spiking the control samples at 0.20, 0.30, and 0.40-μg ml^−1^in triplicate, and samples were processed by the already described method. The mean recovery levels were found to be in the range of 81.32 to 92.13%. The percent relative standard deviation (RSD) was expressed as repeatability of the method. The % RSD ranged from 0.91–1.31% at different spiking levels (Table 1). These results have been reported without applying any correction factors.

**Table 1:** Spiking level and recovery of insecticides from fish muscles.

Spiking levels (μg g^−1^)	Mean recovery* (%) ± SD	Relative standard deviation (%)
0.20	92.13 ± 1.453	1.31
0.30	81.32 ± 2.876	1.23
0.40	86.56 ± 0.753	0.91

* Average of three replications

### 3.2 Insecticide retention in fish muscles

The analysis of fish muscles on HPLC revealed that both chlorpyrifos and dimethoate residues got accumulated in the fish muscles through penetration and showed their traces in the fish body. In range finding test of dimethoate, common carp was exposed to five concentrations viz., 0.5, 1.0, 2.0, 4.0 and 8.0 μg g^−1^, among which the highest percentage of extracted residue i.e. 20.88% was observed in the highest used fortification level of 8.0 μg g^−1^ administered to fishes while as the least recovery of 9.40% was found in the lowest used fortification level of 0.5 μg g^−1^. When compared to the fortification level of the insecticide supplied, the percentage of dimethoate residue in fish muscles was shown to follow first order kinetics, increasing with the insecticide's concentration (Table 2).

**Table 2:** Bioaccumulation of dimethoate in fish muscles exposed to different concentrations of range finding tests.

Level of fortification used in bioassay (μg g^−1^)	Residue penetrated in fish muscles (μgg^−1^)*	Extracted residue from fish muscle (%)	Residue present in the medium (%)
0.5	0.047	9.40	90.60
1.0	0.127	12.70	87.30
2.0	0.262	13.10	86.90
4.0	0.668	16.70	83.30
8.0	1.671	20.88	79.12

* Mean of three replications

Chlorpyrifos was found to accumulate in the muscles more than dimethoate, despite being given in extremely modest doses. Fish were exposed to concentrations of 4.0, 8.0, 16.0, 32.0, and 64.0 ppb during a range-finding test, whereas the median lethal dosage (LC50) of chlorpyrifos was shown to be 3.8 ppb. Only at 32 and 64 ppb, the two highest doses in the range finding test, with recovery percentages of 8.34% and 11.06%, was chlorpyrifos discovered in the fish muscles (Table 3). Chlorpyrifos was also found in the edible portion of common carp that had been exposed to various bioassay concentrations.

**Table 3:** Bioaccumulation of chlorpyrifos in fish muscles exposed to different concentrations of range finding tests.

Level of fortification used in bioassay (ng g^−1^)	Residue penetrated in fish muscles (ng g^−1^) *	Extracted residue from fish muscle (%)	Residue present in the medium (%)
4.0	BDL	BDL	BDL
8.0	BDL	BDL	BDL
16.0	BDL	BDL	BDL
32.0	2.67	8.34	91.66
64.0	7.08	11.06	88.94

### 3.3 Bio-accumulation of insecticides in fishes

At median lethal concentration (LC_50_) values, the recovery of dimethoate from fish muscles was 0.131 μg g^-1^, i.e. 11.90% recovery against the fortification of 1.1 μgg^−1^(Table 4). Residues of the dimethoate in the medium were found to be 88.10%. For rest of the concentrations used in range finding test, chlorpyrifos was found below detection levels. In median lethal concentration of chlorpyrifos, no residues were recovered from the fish muscles and were found below detection levels of HPLC (Table 5).

**Table 4:** Bioaccumulation of dimethoate in fish muscles exposed to median lethal concentration (LC_50_) values.

Level of fortification used in LC_50_values (μg g^-1^)	Residue penetrated in fish muscles LC_50_values (μg g^-1^)*	Extracted residue from fish muscle (%)	Residue present in the medium (%)
1.1	0.131	11.90	88.10

* Mean of three replications

**Table 5:** Bioaccumulation of chlorpyrifos in fish muscles exposed to median lethal concentration (LC_50_) values.

Level of fortification used in LC_50_ values (ng g^-1^)	Residue penetrated in fish muscles LC_50_ values (ng g^-1^)*	Extracted residue from fish muscle (%)	Residue present in the medium (%)
3.8	BDL	BDL	BDL

* Mean of three replications

### 3.4 Bio-concentration Factor

The bio-concentration factor (BCF) was observed increasing progressively with the concentration of insecticides provided to fish. In the concentrations of definitive tests, the bio-concentration factor increased from 0.094 L/g to 0.2088 L/g from low to high concentration (Table 6) in dimethoate. In case of chlorpyrifos, insecticide was not detected except in higher concentrations with BCF of 0.0834 L/g and 0.1106 L/g in 32 ppb and 64 ppb respectively (Table 7). In median lethal concentration (LC_50_) values, bio-concentration value for chlorpyrifos was not found as the insecticide was below detection levels in the fish muscles. In dimethoate, BCF was 11.90 L/g for LC_50_ of 1.1 ppm with 0.131 µg/g of insecticide recovered from fish muscles (Table 8).

**Table 6:** Bio-concentration of dimethoate in fish muscles exposed to the concentration of definitive tests for LC_50_ values.

Amount of insecticide fortified	Insecticide recovered in fish muscles	Bio-concentration factor
0.5	0.047	0.094
1.0	0.127	0.127
2.0	0.262	0.131
4.0	0.668	0.167
8.0	1.671	0.2088

**Table 7:** Bio-concentration of chlorpyrifos in fish muscles exposed to the concentration of definitive tests for LC_50_ values.

Amount of insecticide fortified	Insecticide recovered in fish muscles	Bio-concentration factor
4.0	BDL	BDL
8.0	BDL	BDL
16.0	BDL	BDL
32.0	2.67	0.0834
64.0	7.08	0.1106

BDL= Below detectable level

**Table 8:** Bio-concentration of insecticides in fish muscles exposed to median lethal concentration (LC_50_) values.

Name of the Insecticide	Amount of insecticides fortified (LC_50_)	Insecticide recovered in fish muscles	Bio-concentration factor
Dimethoate	1.1	0.131	11.90
Chlorpyrifos	BDL	BDL	BDL

## 4 Discussion

Fish is an excellent source of not just proteins but also omega-3 polyunsaturated fatty acids, which are beneficial in cardiovascular disease [19]. Among various xenobiotics, insecticides have been found accumulated in fish tissues and their retention has been proven detrimental for the hamlets consuming them. The bioaccumulation of pesticide residues in fish muscles is a crucial component of toxicology because it gives an indication of both the quantity of insecticide-contaminated fish that can be consumed by humans as well as the extent to which the insecticide residues have penetrated into the muscle tissues. The prolonged toxicity of those pesticides with a lipophilic nature (such organochlorines and PCBs) tends to accumulate in the muscles of fish. Organochlorines are lipid soluble and tend to bioaccumulate in biological organism's fat deposits [36]. On the other hand, organophosphorus insecticides are preferred and utilised in agricultural pest control since they are less persistent than organochlorine insecticides [5] and are more likely to break down into less harmful, simpler compounds. This explains why organophosphorous insecticides have largely supplanted organochlorine chemicals as a method of application worldwide. However, it has also been demonstrated that these organophosphorous substances have unfavourable consequences, such as the inhibition of cholinesterase activity in the brain, liver, and muscle of several freshwater teleosts [9].

Two organophosphorous substances were employed in the current investigation to estimate the fatal concentration in *Cyprinus carpio* var. *communis* juveniles. All of the dimethoate concentrations used for the bioassay in this investigation were recovered from the fish muscles. The extracted residue of dimethoate derived from range-finding studies performed on fish muscles was 9.40%, 12.70%, 13.10%, 16.70% and 20.88% at 0.5, 1.0, 2.0, 4.0, and 8.0 0μgg^-1^level of fortification used in bioassay respectively while as for median lethal concentration (LC_50_) of dimethoate, the percentage extraction from fish muscle was 11.90%. Dimethoate tends to breakdown quickly in water while having a low octanol-water partitioning coefficient (log Koc) and a high water-solubility (25 gL-1) [6]. The choice of relatively small fish for the study and the restricted water supply in the aquarium may be responsible for the buildup of dimethoate in the fish tissue at all bioassay doses. As a result, the major routes of entrance dermal absorption through integument and absorption by gills via water had been made possible by the dimethoate's constant availability in vivo. Similar trends for aldrin and parathion accumulation in the liver tissues of *Colisa fasciatus* and *Notopterus* sp. were earlier observed [38]. Due to the greater concentrations employed in the bioassay to calculate LC_50_ values, the significant % recovery of the dimethoate may be explained. Dimethoate was identified in fish muscle tissue and water at levels of 1.30 gkg^-1^ and 4.21 gL^-1^, respectively in *Oreochromis mossambicus* [4]. **Van** *et al.* (2016) similarly revealed the little capacity for bioaccumulation of dimethoate is due to its hydrophilic nature [37]. The efficacy of fish defence systems in reducing the insecticide into excretable components was indicated by the low levels of dimethoate in fish muscle tissues compared to those of the water. Potential toxicity of dimethoate in common carp fingerlings must be constantly evaluated in order to minimise its effects on the food chain. According to the earlier findings of [30], the median fatal amounts of dimethoate are concerning, with a wide range of toxicity among fish. Fukuto [16] noted that *Heteropneustes fossilis* treated to dimethoate displayed greater opercular movement, fatigue, erratic swimming, loss of buoyancy, muscle tetany, and dimming skin colour [5] and these investigations will aid in determining the corrective actions for polluted organisms at the right moment. The poisoning impact of dimethoate on *Clarias* muscle tissue was observed by the boosted rate of protein catabolism as indicated by an increase of amino-transaminases enzyme levels in muscle tissue.

Aquatic organisms are harmed by chlorpyrifos, an insecticide that is often used and permeates most rivers. Remains from the muscles of the fish that had been exposed to chlorpyrifos were also retrieved and analysed. Only the two higher concentrations of range finding tests, i.e. 32 and 64 ng g^-1^, yielded its residues. The residues of chlorpyrifos were below the detection level for various range finding tests and median lethal concentration (LC_50_) values. The structural makeup of the chemical may be the cause of the accumulation of chlorpyrifos at such high concentrations in fish muscles. The benzene ring has three chlorine atoms in the 2-ortho and 1-meta locations. As a result, the compound more closely resembles an organochlorine class compound than an organophosphate group compound. In other words, chlorpyrifos is more lipophilic than other organophosphates and tends to accumulate in fish tissue at higher concentrations than any other pesticide in its class. According to Faria et al., [14] the reduction in AChE activity was brought on by a progressive rise in chlorpyrifos-oxon-concentration. The muscle structure and function, including a decrease in motor activity, were altered in the examined embryos, which also revealed a shortening of the trunk and axial slow muscle fibres. A total paralysis of the embryo occurred at the greatest CPO concentration. The majority of the effects of organophosphates on humans were effectively thrived by these symptoms. After 21 days, the muscle of *C. gariepinus* treated to 28 gL^-1^chlorpyrifos showed a decrease in amino acid levels [20]. Additionally, it has a very low water solubility of 0.4–2.0 mgL^-1^ and a very high Octanol-water partitioning coefficient (log K_OW_ = 5.2) [25] which suggests that it will continue to float on the water, making it readily accessible to fishes in large quantities. This explains why larger concentrations of chlorpyrifos enter into fish muscles, as was seen in the current investigation. Our findings concur with those made by Mahboob et al. [22], who noted the accumulation of several pesticide residues in *Cirrhinus mrigala* taken from Pakistani natural water bodies and rearing facilities. They reported that among all the pesticides, dimethoate had the greatest concentration (0.160.001 ppm), which was found in the muscles of the fish. According to Miranda et al. [24], the muscle tissue of the freshwater fish *Hopliasala baricus* underwent histological changes as a result of exposure to PCBs and chlorinated insecticides. A two-week chlorpyrifos treatment resulted in increased degeneration, necrosis, and localised haemorrhage in *Labeo rohita* liver tissue [13]. Common carp (*Cyprinus carpio*) liver and gill tissue were damaged by low concentrations of chlorpyrifos (11.6–116 gL^-1^). The liver tissue displayed different degrees of hydropic degeneration, vacuolization, pyknotic nuclei, and fatty infiltration. The gill tissue had different degrees of oedema, telangiectasis, and epithelial hypertrophy [39]. Fish play a significant part in the food chain, therefore studying how hazardous insecticides like chlorpyrifos affect fish can be a useful diagnostic tool for determining how insecticides harm human health. *Labeo rohita* when exposed to insecticide (0.1891 ppm) for two days, were found to have no significant shift in proteins [31]. The brain experienced the greatest and most significant depletion, whereas the muscle experienced the least. The amount of cholesterol dropped during the time of exposure period; it is possible that dimethoate damages the liver capacity to store cholesterol, blocks the ovary steroidogenesis-related enzyme system, and causes generalised harm [17]. Dimethoate exposure in *Channa punctatus* resulted in cytoplasm vacuolization and cytoplasm granules (Annes, 1978). Dimethoate had an effect on the oxygen consumption of *Tilapia mossambica* at all concentrations tested. The observed increase in oxygen use by the entire animal could be due to respiratory distress caused by oxidative metabolic impairment [34]. Increased chlorpyrifos concentration and treatment time resulted in a greater mortality rate in *Tilapia guineensis* [8]. Lipid peroxidation, oxidative stress, and metabolic changes in several tissues, such as the liver, gills, and kidney, may have resulted from free radicals formed during the detoxification of dimethoate and Bacilar fertiliser.

According to reports, fish exposed to organophosphate insecticides exhibit anomalous behaviour and have a higher death rate as a result of metabolic changes connected to toxicants [11]. Chlorpyrifos has been proven to have a significant effect on the growth of *O*. *niloticus* [23]. A significant difference in the haemoglobin concentration and white blood cells of *O. niloticus* was observed after 30 days of exposure to chlorpyrifos [2]. Fish subjected to organophosphate insecticides have reportedly shown aberrant behaviour and a higher death rate as a result of metabolic changes connected to toxicants. Dembélé et al., ($year$) [11]. In *Tandanus tandanu* treated with chlorpyrifos, negative growth problems were found [18]. The liver served as the primary organ for metabolite detoxification, and its histology would serve as an excellent biomarker for demonstrating the impact of toxicant at the cellular level. Cuevas et al., ($year$) [10]. The body requires more oxygen when toxicants enter through the gills. Monitoring any dangerous stress in the aquatic environment as a result is an important indicator [27]. Thus, the retention of organophosphates in fish poses a significant concern for both aquatic ecosystems and human health. The bioaccumulation of these toxic chemicals can lead to long-term ecological imbalances and potential health risks for those consuming contaminated fish. Effective measures are essential to mitigate their impact, including strict regulations and sustainable agricultural practices to reduce organophosphate usage.

## 5 Conclusion

This study indicates that insecticides have potential to penetrate in the muscle of *Cyprinus carpio* var. *communis*. Exposure of fishes to these xenobiotics in natural conditions could be envisaged from the present study. It is thus recommended to strategize the lake management practices as far as insecticide removal is concerned. Rivers, lakes, saltwater, and rainwater have all been shown to contain organophosphates. Therefore, it is essential to create more effective legislation limiting organophosphate use, pollution emission limitations, and disposal procedures, as well as to define environmental safety standards in various water bodies, in order to safeguard aquatic ecosystems. It is critical to gain a better knowledge of organophosphates eco-toxicity, in order to restrict the spread of toxicity in the aquatic ecosystems and reduce health risks to the humans.

## Funding

Nil.

## CRediT authorship contribution statement

**Jan Ishrat:** Methodology, Investigation, Data curation. **Sherwani Asma:** Resources, Investigation, Data curation, Conceptualization. **Khan Sameena:** Methodology, Investigation, Formal analysis. **Siddiqui Uzma:** Methodology, Investigation, Formal analysis, Data curation. **Abubakr Adnan:** Methodology, Investigation, Formal analysis, Conceptualization. **Mukhtar Malik:** Validation, Software, Investigation, Conceptualization. **Balkhi Masood:** Writing – original draft, Validation, Investigation, Conceptualization. **Qayoom Imtiyaz:** Writing – original draft, Conceptualization. **MASTINU ANDREA:** Writing – review & editing, Supervision, Project administration, Funding acquisition. **Sayyed Riyazali:** Writing – review & editing, Writing – original draft, Formal analysis, Data curation, Conceptualization.

## Declaration of Competing Interest

The authors declare the following financial interests/personal relationships which may be considered as potential competing interests: Andrea Mastinu reports article publishing charges was provided by University of Brescia. If there are other authors, they declare that they have no known competing financial interests or personal relationships that could have appeared to influence the work reported in this paper.

## Data Availability

Data will be made available on request.
